# Bone marrow transplantation in AML, and socioeconomic class: a UK population-based cohort study

**DOI:** 10.1186/1471-2407-10-514

**Published:** 2010-09-28

**Authors:** Fatima Bhayat, Emma Das-Gupta, Richard Hubbard

**Affiliations:** 1 Division of Epidemiology and Public Health, University of Nottingham, Clinical Sciences Building, Nottingham City Hospital, Hucknall Road, Nottingham, NG5 1PB, UK; 2 Clinical Haematologist, Centre for Clinical Haematology, Nottingham University Hospitals, City Hospital Campus, Hucknall Road, Nottingham, NG5 1PB, UK; 3 Professor Clinical Epidemiology, Division of Epidemiology and Public Health, University of Nottingham, Clinical Sciences Building, Nottingham City Hospital, Hucknall Road, Nottingham, NG5 1PB, UK

## Abstract

**Background:**

We have previously shown that in the UK mortality in people with Acute Myeloid Leukaemia (AML) was nearly 50% greater among the most socio-economically deprived. The aim of this study was to determine whether AML patients from lower socioeconomic classes had a lower chance of receiving a bone marrow transplant.

**Methods:**

Using Hospital Episode Statistics (HES) data, we identified all incident cases of AML admitted to UK hospitals between 1998 and 2007. We calculated the number of bone marrow transplantations undertaken in AML patients, stratifying our results by gender, age at diagnosis, year of diagnosis, degree of socioeconomic deprivation and co-morbidity. We used logistic regression to calculate odds ratios for bone marrow transplantation, adjusting for gender, age at diagnosis, year of diagnosis, degree of socioeconomic deprivation and co-morbidity score.

**Results:**

We identified a total of 23 910 incident cases of AML over this 10-year time period, of whom 1 140 (4.8%) underwent BMT. Bone marrow transplantation declined with increasing socioeconomic deprivation (p for trend < 0.001) such that people in the most deprived socioeconomic quintile were 40% less likely to have a transplant than those in the most advantaged group (Odds Ratio 0.60, 95% confidence interval 0.49, 0.73), even after adjusting for gender, age at diagnosis, year of diagnosis and co-morbidity.

**Conclusion:**

This large cohort study demonstrates that AML patients from lower socioeconomic classes are less likely to undergo bone marrow transplantation than their better off counter-parts.

## Background

We have previously shown that while the incidence of Acute Myeloid Leukaemia (AML) in the UK is similar across the spectrum of social class, mortality was nearly 50% greater among the most socioeconomically deprived patients than among the most advantaged[1]. We postulated that this difference in survival might be due to different patterns of treatment, such as bone marrow transplantation (BMT), and/or the presence of greater co-morbidity, such as heart disease and COPD, among patients from lower socioeconomic classes. Previous studies have shown that people from more deprived background are less likely to receive chemotherapy and radiotherapy for a range of cancers including breast[2], lung[3] and colorectal[3,4] cancers.

In this study we have used Hospital Episode Statistics (HES) data to determine whether people with AML from lower socioeconomic classes are less likely to receive bone marrow transplantation than those from higher socioeconomic classes. We also wished to determine whether any difference in the use of BMT found was due to greater co-morbidity among more deprived patients than among the better off.

## Methods

We used anonymised hospital episode statistics (HES) data[5] for this study. These are record-level data administered by The NHS Information Centre for Health and Social Care, on behalf of the Secretary of State for Health. Data are extracted from routine data flows between healthcare providers and commissioners and used to populate the HES datasets. The admitted patient dataset, which includes inpatient and day-case records, was used in this research. Cumulative data are extracted quarterly for this dataset and it is also updated annually. The information contained within HES includes patient demographics such as date of birth, gender and region of residence, details of diagnoses and treatments received, as well as administrative details such as admission and discharge dates, as well as the place patients were treated (NHS Trust or independent sector hospital, for example).

We identified all incident cases of AML admitted to UK hospitals between 1998 and 2007, including those admitted as day-cases. We also identified all admissions for co-existing medical diagnoses recorded in HES for these patients, along with all procedures, including bone marrow transplantation, which they underwent during all of their admissions over this period. Bone marrow transplantation for the purposes of this study means allogeneic stem cell transplantation with cells harvested from peripheral blood or bone marrow.

We calculated the number of bone marrow transplantations undertaken in AML patients, stratifying our results by gender, age at diagnosis, year of diagnosis, degree of socioeconomic deprivation and co-morbidity. We used Townsend Score as the measure of socioeconomic deprivation, which is derived from 2001 census output data and divided into quintiles, with 1 being the least deprived group and 5 being the most deprived. The score is based on a combination of four variables namely: unemployment; car ownership; home ownership and overcrowding, which produce a ranking of a particular small homogenous socio-geographic area of about 150 homes[6].

We used co-morbid illness data recorded on admission to assign a co-morbidity score to each patient using the Charlson Co-morbidity Index[7]. This scoring takes into account the presence of 19 different medical disease groups, each of which carries a weight ranging from 1 to 6, depending on the relative risk of death within 12 months associated with the presence of the particular disease group. The Charlson Co-morbidity Index has been found to have good reliability and to correlate well with mortality[7].

We used logistic regression to calculate odds ratios for bone marrow transplantation, adjusting for gender, age at diagnosis, year of diagnosis, degree of socioeconomic deprivation and co-morbidity score. All data management and statistical analyses were conducted using STATAv.10.0.

## Results

We identified a total of 23 910 incident cases of AML over this 10-year time period, of whom 1 140 (4.8%) underwent BMT. The numbers of bone marrow transplants performed across various strata in AML are shown in Table 1. A similar proportion of men and women had BMT, about 5%. The frequency of bone marrow transplantation decreased with increasing age at diagnosis, with only 3 transplants recorded in those aged 71 or older. A smaller percentage of patients from lower socioeconomic classes had transplants than those from higher socioeconomic classes. The relationship between socioeconomic class and co-morbidity is shown in Table 2. Between 68 and 70% of AML patients have a Charlson Co-morbidity Score of 0, 14 -16% have a Score of 1, and 16% have a Score of 2 or more, with no significant differences across socio-economic classes in any of the co-morbidity categories.

**Table 1:** Numbers of Bone Marrow Transplants

	BMT		TOTAL
	**No**	**Yes (%)**	

**Overall**	22 770	1 140 (4.8)	23 910
			
Gender			
Males	12 695	614 (4.6)	13 309
Females	10 008	525 (5.0)	10 533
Not Known	67	1	68
**Age at Diagnosis**			
Up to 30	2 909	417(13)	3 326
31 to 40	1 463	213(13)	1 676
41 to 50	1 912	222(10)	2 134
51 to 60	2 947	237(7)	3 184
61 to 70	4 523	48(1)	4 571
71 and older	9 013	3(0)	9 016
**Year of Diagnosis**			
1997 to 1999	4 730	262 (5.2)	4 992
2000 to 2001	4 873	290 (5.6)	5 163
2002 to 2003	4 913	266 (5.1)	5 179
2004 to 2007	8 254	322 (3.8)	8 576
**Townsend Score**			
1	4 433	265 (5.6)	4 698
2	4 495	226 (5.6)	4 721
3	4 179	190 (4.3)	4 396
4	3 968	207 (4.9)	4 175
5	3 735	186 (4.7)	3 921
No Record	1 960	66 (3.3)	2 026
			
**Charlson Co-morbidity Score**			
0	16 174	855 (5.0)	17 029
1	3 238	108 (3.2)	3 346
2 or more	3 358	177 (5.0)	3 535

**Table 2:** Proportion of AML patients in each category of Charlson Score by Social Class

	Charlson Score					
	**0**		**1**		**2 or more**	

	**%**	**95% C.I**.	**%**	**95% C.I**.	**%**	**95% C.I**.

**Townsend Score**						
**1**	70	69 - 72	14	13 - 15	16	15 - 17
**2**	68	67 - 70	16	14 - 17	16	15 - 17
**3**	69	68 - 71	15	14 - 16	16	15 - 17
**4**	69	68 - 70	15	14 - 16	16	15 - 17
**5**	68	67 - 70	16	15 - 17	16	15 - 17
**No Record**	94	93 - 95	3	2 - 4	2	2 -3

Odds Ratios for bone marrow transplantation in AML are shown in Table 3. Bone marrow transplantation declines with increasing age at diagnosis after the age of 30 (p for trend < 0.001), as well as with increasing socioeconomic deprivation (p for trend < 0.001). Patients with AML in the most deprived socioeconomic quintile are 40% less likely to have a bone marrow transplantation than those in the most advantaged socioeconomic class (OR 0.60, p < 0.001, 95% C.I. 0.49, 0.73), even after adjusting for gender, age at diagnosis, year of diagnosis and co-morbidity. No statistically significant interaction was found between Townsend score and age, or year of diagnosis. There was, however, statistically significant interaction (p = 0.01) between Townsend score and gender, with greater differences in odds ratios across the socioeconomic class gradient among women than men. Results of logistic regression (Odds Ratios) stratified by gender are shown in Table 4.

**Table 3:** Odds Ratios for Bone Marrow Transplantation

	Uni-variate Analyses			Multi-variate Analyses**		
	**O. R**.	P>z	**95% C.I**.	**O. R**.	P>z	**95% C.I**.
**Gender**						
Males	1	-	-	1	-	-
Females	1.01	0.82	0.92 - 1.11	0.91	0.03	0.83 - 0.99
						
**Age at Diagnosis**						
Up to 30	1	-	-	1	-	-
31 to 40	1.02	0.86	0.85 - 1.21	0.86	0.10	0.72 - 1.03
41 to 50	0.82	0.02	0.68 - 0.96	0.65	< 0.001	0.54 - 0.77
51 to 60	0.56	< 0.001	0.47 - 0.66	0.40	< 0.001	0.34 - 0.48
61 to 70	0.07	< 0.001	0.05 - 0.10	0.05	< 0.001	0.04 - 0.07
71 and older	0.00	< 0.001	0.00 - 0.01	0.00	< 0.001	0.00 - 0.00
						
**Year of Diagnosis**						
1997 to 1999	1	-	-	1	-	-
2000 to 2001	1.07	0.41	0.90 - 1.28	1.12	0.21	0.94 - 1.34
2002 to 2003	0.98	0.80	0.82 - 1.16	1.11	0.28	0.92 - 1.33
2004 to 2007	0.70	< 0.001	0.60 - 0.83	0.84	0.05	0.70 - 1.00
						
**Townsend Score**						
1	1	-	-	1	-	-
2	0.84	0.06	0.70 - 1.01	0.85	0.10	0.70 - 1.03
3	0.76	0.01	0.63 - 0.92	0.76	0.01	0.63 - 0.93
4	0.87	0.15	0.72 - 1.05	0.78	0.01	0.64 - 0.95
5	0.83	0.06 *p = 0.1	0.69 - 1.01	0.60	< 0.001 *p < 0.001	0.49 - 0.73
No Record	0.56	< 0.001	0.43 - 0.74	0.18	< 0.001	0.14 - 0.24
						
**Charlson Co-morbidity Score**						
0	1	-	-	1	-	-
1	0.63	< 0.001	0.51 - 0.77	1.03	0.80	0.83 - 1.27
2 or more	0.99	0.97	0.84 - 1.18	1.58	< 0.001	1.33 - 1.89

*p = test for trend across Townsend Scores 1 to 5.

**Adjusted for gender, age at diagnosis, year of diagnosis Townsend Score and co-morbidity.

**Table 4:** Odds Ratios for Bone Marrow Transplantation Stratified by Gender

	O. R.**	P>z	**95% C.I**.
**MALES**			
			
**Townsend Score**			
1	1	-	-
2	0.83	0.16	0.63 - 1.08
3	0.77	0.07	0.58 - 1.02
4	0.83	0.17	0.63 - 1.08
5	0.68	0.00 *p = 0.01	0.52 - 0.88
No Record	0.18	< 0.01	0.12 - 0.26
			
**FEMALES**			
			
**Townsend Score**			
1	1	-	-
2	0.86	0.29	0.65 - 1.14
3	0.74	0.04	0.56 - 0.98
4	0.73	0.03	0.54 - 0.97
5	0.51	< 0.01 *p < 0.01	0.38 - 0.70
No Record	0.21	< 0.01	0.14 - 0.34

**Adjusted for age at diagnosis, year of diagnosis and Charlson Co-morbidity Score.

*p = test for trend across Townsend Scores 1 to 5.

## Discussion

This study has shown that people with AML from more deprived socioeconomic classes are less likely to undergo bone marrow transplantation than their counter-parts from more advantaged social classes, even after adjusting for the presence of recorded co-existing disease.

The main strength of this study is the large size of the study population. We were able to study over 23 000 incident cases of AML in the UK using data derived from hospital records. Data also included the co-existing medical conditions of AML patients, which allowed us to adjust for co-morbidity. One drawback, however, was that our data on co-morbidity was based on hospital admission records for those conditions. This means that whilst we should have captured the most severe co-morbid illness we will have missed more minor disease. As a result it is possible that there is some residual confounding by co-morbid illness in this study and that some of the gradient in socioeconomic status and access to bone marrow transplantation is still due to co-morbid disease.

By using routinely collected and available data rather than questionnaires or interviews, we have eliminated any bias in the reporting of socioeconomic class and any social class bias in participation in the study. Bone marrow transplantation recording is also likely to be accurate in hospital records given the highly specialised nature of the procedure. It seems likely to us therefore that the validity of this information is good.

One potential weakness of this study is that we were unable to adjust for the cytogenetic risk group of our AML patients. Based on cytogenetics at presentation, AML patients are classified into good, intermediate, and adverse risk groups, each with very different long term outcomes[8]. Good risk patients in their first remission are not transplanted, whereas adverse risk patients are almost always transplanted (subject to fitness and donor availability). Any bias introduced by this in our results, however, is likely to apply across all social classes, unless patients from lower social classes are more likely to be in the good risk group than those from higher socioeconomic classes, for which there is no evidence. Although there is evidence that patients from lower social classes present later with disease symptoms in general, it is less likely that late presentation is an important factor in AML survival given the acute presentation of the disease and its relatively poor prognosis. For intermediate risk patients, however, it is possible that later presentation may have an impact on the treatment administered and its outcome.

The accuracy of social class classification is imperfect given that Townsend Score is not an individual measure of deprivation. This will have introduced a non-differential bias into our results, if any, i.e. both patients who had had a bone marrow transplant and those who had not will have been similarly affected. Such a bias will have moved odds ratios closer to '1'. It seems then that if we had been able to perfectly adjust for socio-economic deprivation, our results may have shown an even greater class bias.

The validity of co-morbidity recorded in HES data may also be imperfect. Any inaccuracies would, however, apply equally across social class strata and so is unlikely to have introduced bias into these results. Furthermore these results showed no difference in recorded comorbidity across the social classes. Residual confounding cannot be ruled out completely, however, since only comorbidity recorded in the hospital episode data have been taken into account. Other comorbidities not related to hospital admission or not recorded during the admission may have existed which would have resulted in incomplete adjustment for comorbidity.

To our knowledge no studies examining the association between bone marrow transplantation and socioeconomic class have previously been published. Studies have, however, examined the associations between social class and chemotherapy in a number of cancers. Several studies found that lower socioeconomic class predicted under-use of chemotherapy in colorectal cancer (CRC)[3,4], breast cancer[2] and lung cancer[3]. Two North American studies found that low socioeconomic status was associated with under-use of adjuvant chemotherapy in both breast[2] and colorectal cancers[4], and postulated that this was in part due to a combination of poor access to care, financial barriers and physicians' assumptions and biases regarding patients from lower socioeconomic classes, such as the availability of social and monetary support, their expectations of treatment and their likely compliance with treatment, for example. A further study concluded that lower incomes, absent or limited insurance cover and poorer education reduced access to high-quality adjuvant chemotherapy, which in turn reduced survival in breast cancer[9].

In the UK, a Scottish study showed that patients from the poorest deprivation quintile were less likely to receive chemotherapy for lung cancer and colorectal cancer than the most advantaged patients after adjusting for age, tumour stage at diagnosis, health authority and distance from oncology centre[3]. Delay between referral and treatment was similar across all social classes and so did not explain the findings. Although this study did not adjust for comorbidity, another Scottish study which had done so also found poorer survival in colorectal cancer patients from the most deprived socioeconomic quintiles, in a study population which showed no correlation between socioeconomic deprivation and co-morbidity[10]. The findings of these studies, in the UK healthcare setting where access to treatment is equal and free, suggest that decision-making (by both physicians and patients) regarding chemotherapy may be influenced by non-clinical factors.

## Conclusions

This large cohort study demonstrates that people with AML from lower socioeconomic classes in the UK are less likely to undergo bone marrow transplantation than their better off counter-parts. Further work is now required to fully understand the reasons for this finding and to ensure equal access to treatment for people from all backgrounds.

## Competing interests

The authors declare that they have no competing interests.

## Authors' contributions

FB conducted the data management, performed the statistical analyses and drafted the manuscript. EDG contributed to revising the manuscript for intellectual content. RH conceived of the study, acquired the data and edited the manuscript. All authors read and approved the final manuscript.

## Pre-publication history

The pre-publication history for this paper can be accessed here:

http://www.biomedcentral.com/1471-2407/10/514/prepub
